# Simultaneous three-port thoracoscopic surgery for bilateral lung cancers with a pulmonary vein anomaly: A case report

**DOI:** 10.1016/j.ijscr.2018.11.054

**Published:** 2018-11-24

**Authors:** Miho Akabane, Tadasu Kohno, Sakashi Fujimori, Naoko Kimura, Souichirou Suzuki, Mitsuaki Kawashima, Shinichirou Kikunaga

**Affiliations:** Department of Thoracic Surgery, Respiratory Centre, Toranomon Hospital, 2-2-2 Toranomon, Minato-ku, Tokyo, 105-8470, Japan

**Keywords:** PAPVC, partial anomalous pulmonary venous connection, SCC, squamous cell carcinoma, PET, positron emission tomography (PET), SUV, standardized uptake value, SVC, superior vena cava, Simultaneous thoracoscopic resection, Bilateral lung cancers, Partial anomalous pulmonary venous connection

## Abstract

**Introduction:**

Simultaneous resection of bilateral lung cancers is technically challenging but may be preferable to a staged procedure in patients with a partial anomalous pulmonary venous connection (PAPVC) in an affected lobe. We performed single-stage resection of bilateral lung cancers in a patient with a PAPVC.

**Presentation of case:**

A 73-year-old man was diagnosed as having bilateral lung cancers (right, cT3N1M0, stage IIIA and left, cT2aN0M0, stage IB). Left upper trisegmentectomy was performed, followed by right upper lobectomy with deep wedge bronchoplasty. A PAPVC was found incidentally in the affected right upper lobe and successfully divided. The postoperative course was uneventful and he commenced chemoradiotherapy.

**Discussion:**

Resection of the PAPVC, which was located in the same lobe as the lung cancer, would have mitigated load increase in the right heart and may have alleviated the adverse effects of bilateral lung resection. Moreover, the single-stage procedure likely shortened the overall duration of treatment.

**Conclusion:**

Single-stage bilateral thoracoscopic resection may have advantages over staged procedures in some patients with PAPVCs.

## Highlights

• We successfully performed thoracoscopic simultaneous resection of bilateral lung cancers with a PAPVC.
• A single-stage procedure can achieve hemodynamic improvement for some patients with a PAPVC.
• A single-stage procedure can reduce the duration of treatment.
• A single-stage procedure of bilateral lung cancers is a feasible option for some patients with a PAPVC.
• This is the first report regarding concomitant thoracoscopic resection of bilateral lung cancers and a PAPVC.

## 1 Introduction

Synchronous bilateral lung tumours are sometimes excised in a single-stage procedure. However, the right heart pressure may increase, depending on the amount of resected lung parenchyma [1,2]. Partial anomalous pulmonary venous connections (PAPVCs) are characterized by abnormal drainage of some of the pulmonary veins into the right heart. In this report, we describe a case of successful simultaneous thoracoscopic resection of bilateral lung cancers in a patient with a PAPVC.

This case report has been written in line with the SCARE guidelines [3].

## 2 Presentation of case

**Fig. 1:**
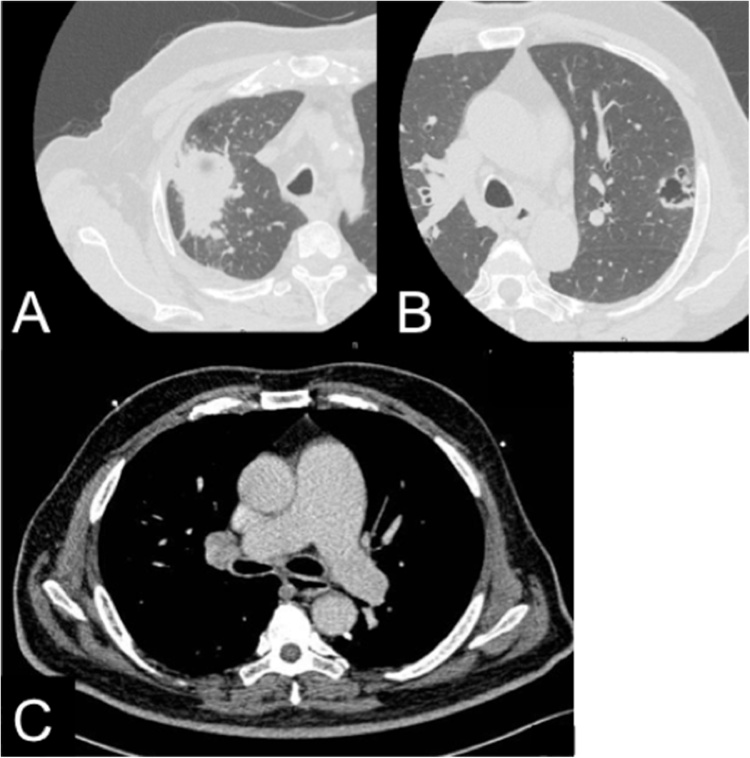
(A) Thoracic computed tomographic scan showing a 5.2 × 4-cm mass lesion in the right lung. (B) A 3.1 × 2.5-cm cavitating lesion in the left upper lobe. (C) Contrast-enhanced computed tomography image showing an enlarged hilar lymph node.

**Fig. 2:**
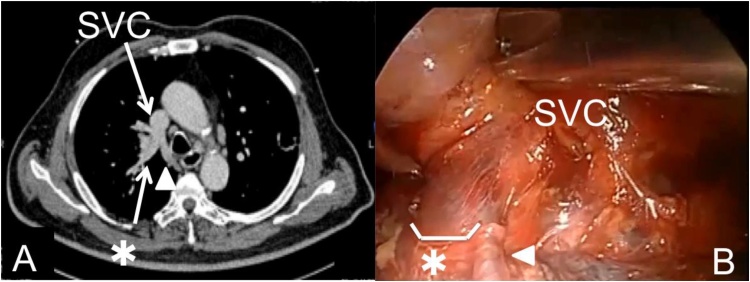
(A) Thoracic computed tomography image showing the right upper pulmonary vein draining into the SVC. (B) Intraoperative photograph showing the PAPVC. Asterisk (*): PAPVC draining into SVC, arrowhead: azygos vein. PAPVC: partial anomalous pulmonary venous connection, SVC: superior vena cava.

A 73-year-old Indian man with a history of heavy smoking was found to have bilateral upper lobe lung masses on computed tomography (CT) during a health check-up. He had no remarkable medical, drug, or family history. His CT scan showed a 5.2 × 4-cm solid mass in the right upper lobe and a 3.1 × 2.5-cm cavitating mass in the left upper lobe. A biopsy of the right lesion resulted in a diagnosis of squamous cell carcinoma (SCC). Positron emission tomography (PET) CT showed significant accumulation with maximum standardized uptake value (SUVmax) 12.5 in the right lesion and 7.5 in the left lesion. Right hilar lymph nodes revealed low accumulation (SUVmax 3.4). The patient was suspected of having bilateral lung cancer (right, cT3N1M0, stage IIIA and left, cT2aN0M0, stage IB), and referred to our institute. Pulmonary function examination showed a forced expiratory volume of 2.4 L/s (83% of the predicted volume) and a forced vital capacity of 3.29 L (92% of the predicted volume). His vital capacity was 3.31 L. Echocardiography revealed mild pulmonary hypertension with tricuspid regurgitation and a pressure gradient of 30 mmHg. His brain natriuretic peptide was 27.2 pg/mL; there were no other blood test abnormalities. The predicted forced expiratory volume in 1 s after right upper lobectomy and left upper trisegmentectomy was 1.71 L, indicating that the patient would tolerate single-stage surgery. The entire procedure was performed thoracoscopically (Fig. 1, Fig. 2).

First, the patient was placed in a right recumbent position and the left side surgery performed through three-port incisions, as described previously [4]. Wedge resection of the left upper lobe was performed and the histological diagnosis of SCC confirmed, following which left trisegmentectomy and upper mediastinal lymph node dissection were carried out. Next, the patient was placed in a left recumbent position and the right side surgery performed via three incisions in the same manner as on the left side. Intraoperatively, it was noticed that the right upper pulmonary vein drained into superior vena cava (SVC); this was recognized as a PAPVC. Because the PAPVC was located only in the affected right upper lobe, reconstruction of the pulmonary veins was unnecessary. The PAPVC was divided using a vascular stapling device and right upper lobectomy and mediastinal lymph node dissection carried out. Because the hilar lymph node around the right upper bronchus was enlarged, deep wedge bronchoplasty was performed. The bronchial surgical margin was negative on an intraoperative frozen section. The bronchial stump was reinforced with the right lobe of the thymus. The operation took 402 min, with 193 mL of blood loss.

The final pathologic diagnosis was bilateral primary lung SCC; the right tumour comprising a keratinizing SCC with lymphatic and vascular permeation (ly1, v1) (pT3N2M0, stage IIIB with #4R and #7 nodes positive) and the left a vaguely keratinizing SCC (pT2aN0M0, stage IB with ly1, v1).

The patient’s postoperative recovery was uneventful and he was discharged on postoperative day 17. He was satisfied with the effect of the treatment he received. He started receiving chemotherapy (carboplatin and vinorelbine) and radiotherapy in another hospital three months after his discharge.

## 3 Discussion

Synchronous multiple lung tumours are rare. Tsunezuka et al. reported that 1.9% of 1906 patients undergoing surgery in their hospital between 1973 and 2001 had bilateral multiple primary lung cancers [5]. Some authors have reported performing single-stage surgery for bilateral multiple lung cancers. In our institute, 2338 patients underwent thoracoscopic surgery for primary lung cancer between 2005 and 2016. Single-stage bilateral thoracoscopic resections were performed in 125 of these patients [2]. However, this procedure is highly invasive and may increase right heart load, depending on the amount of resected lung parenchyma [1]. The greatest disadvantage of staged resection is the lengthy duration of treatment. Although the second procedure is generally planned for about one month after the first one, there is no assurance that the postoperative course will run smoothly. In the present patient, single-stage bilateral thoracoscopic resection was a feasible option and his postoperative course was uneventful.

PAPVC is a rare anomaly that occurs in about 0.4% to 0.7% of the population according to autopsy data [6]. It is characteristically asymptomatic. When a PAPVC is detected along with lung cancer, it may complicate the surgical strategy [7]. If the PAPVC is in the lobe to be resected, right heart failure is not a concern, whereas if the PAPVC is in the remaining lobe, resection could lead to increased shunt flow, resulting in right heart failure [8]. Some authors have suggested that, provided the pulmonary-to-systemic flow ratio (Qp/Qs) is greater than 1.5, PAPVCs should be surgically treated before lung resection [8]. Radiographic detection of vascular anomalies prior to undertaking lung cancer surgery is desirable; however unexpected PAPVCs are often discovered intraoperatively [8].

In the present case, because the PAPVC was located in the same lobe as the lung cancer, it could be divided without venous reconstruction. Resection of the PAPVC should have reduced the increase in right heart preload caused by a left to right shunt and may have contributed to improving the pulmonary artery pressure. The patient had no evidence of right heart failure postoperatively. We speculate that resection of PAPVCs may be helpful in reducing the deterioration in hemodynamic status associated with bilateral lung resections.

To the best of our knowledge, this is the first report of concomitant thoracoscopic resection of bilateral lung cancers and a PAPVC. We think this case is of remarkable pedagogic value because of our conclusion that resection of the PAPVC would have mitigated load increase in the right heart and may have alleviated the adverse effects of bilateral lung resection; additionally, the single-stage procedure may have shortened the overall duration of treatment compared with a staged procedure [9,10].

## 4 Conclusions

After careful preoperative assessment, single-stage bilateral thoracoscopic resection is a feasible surgical option in some patients with PAPVC.

## Conflicts of interest

The authors have no conflicts of interest Ethic approval not required for the publication of this manuscript.

## Sources of funding

None.

## Ethical approval

The study of a case report is exempt from ethnical approval in my institution.

## Consent

Informed and written consent was obtained from the patient for publication of this case and accompanying images.

## Author contribution

Miho Akabane wrote the manuscript. Tadasu Kohno contributed to the operation and follow-up. Souichirou Suzuki and Mitsuaki Kawashima contributed in its design and coordination and helped to draft the manuscript. All authors read and approved the final manuscript. We thank Dr Trish Reynolds, MBBS, FRACP, from Edanz Group (www.edanzediting.com/ac) for editing a draft of this manuscript.

## Registration of research studies

None.

## Guarantor

Tadasu Kohno.
